# A Field Evaluation of an External and Neutrally Buoyant Acoustic Transmitter for Juvenile Salmon: Implications for Estimating Hydroturbine Passage Survival

**DOI:** 10.1371/journal.pone.0077744

**Published:** 2013-10-25

**Authors:** Richard S. Brown, Z. Daniel Deng, Katrina V. Cook, Brett D. Pflugrath, Xinya Li, Tao Fu, Jayson J. Martinez, Huidong Li, Bradly A. Trumbo, Martin L. Ahmann, Adam G. Seaburg

**Affiliations:** 1 Pacific Northwest National Laboratory, Ecology Group, Richland, Washington, United States of America; 2 Pacific Northwest National Laboratory, Hydrology Group, Richland, Washington, United States of America; 3 US Army Corps of Engineers, Walla Walla District, Walla Walla, Washington, United States of America; 4 University of Washington, School of Aquatic and Fishery Sciences, Seattle, Washington, United States of America; Manchester University, United Kingdom

## Abstract

Turbine-passed fish are exposed to rapid decreases in pressure which can cause barotrauma. The presence of an implanted telemetry tag increases the likelihood of injury or death from exposure to pressure changes, thus potentially biasing studies evaluating survival of turbine-passed fish. Therefore, a neutrally buoyant externally attached tag was developed to eliminate this bias in turbine passage studies. This new tag was designed not to add excess mass in water or take up space in the coelom, having an effective tag burden of zero with the goal of reducing pressure related biases to turbine survival studies. To determine if this new tag affects fish performance or susceptibility to predation, it was evaluated in the field relative to internally implanted acoustic transmitters (JSATS; Juvenile Salmon Acoustic Telemetry System) used widely for survival studies of juvenile salmonids. Survival and travel time through the study reach was compared between fish with either tag type in an area of high predation in the Snake and Columbia rivers, Washington. An additional group of fish affixed with neutrally-buoyant dummy external tags were implanted with passive integrated transponder (PIT) tags and recovered further downstream to assess external tag retention and injury. There were no significant differences in survival to the first detection site, 12 river kilometers (rkm) downstream of release. Travel times were also similar between groups. Conversely, externally-tagged fish had reduced survival (or elevated tag loss) to the second detection site, 65 rkm downstream. In addition, the retention study revealed that tag loss was first observed in fish recaptured approximately 9 days after release. Results suggest that this new tag may be viable for short term (<8 days) single-dam turbine-passage studies and under these situations, may alleviate the turbine passage-related bias encountered when using internal tags, however further research is needed to confirm this.

## Introduction

All hydro turbine-passed fish are exposed to a rapid decrease in pressure. Rapid pressure changes can result in injuries such as swim bladder rupture, exophthalmia, emboli, and hemorrhaging [1]–[5]. These types of injuries due to pressure change are known as barotrauma.

The movement and survival of turbine-passed fish is typically evaluated using implanted telemetry tags (e.g., acoustic, radio, inductive [such as passive integrated transponders or PIT tags]) [6], [7]. However, a telemetry tag implanted inside the coelom of small fish such as juvenile salmon could increase the likelihood of injury or mortality following exposure to rapid decompression [8]. Using experimental pressure scenarios that simulate turbine passage, Carlson et al. [8] determined that the probability of mortal injury (mortality or injury highly associated with mortality) varies with tag burden (i.e., the weight of a transmitter relative to the weight of the fish). Fish implanted with larger transmitters, or having a higher tag burden, have a higher probability of mortal injury during simulated turbine passage. The excess mass (difference between the gravitational and buoyant forces acting on an object or in this case weight in water) of an implanted tag has been shown to lead to an increase in swim bladder volume; that is, a fish increases displacement to balance the additional mass [9], [10]. This increased volume of gas in the swim bladder then leads to a higher likelihood that fish will suffer barotrauma when the gas expands during rapid decompression associated with turbine passage [11]–[12].

Thus, estimates of turbine passage survival obtained using tags implanted into the coelom of fish may be inaccurate and biased toward higher mortality rates. Accurate and precise assessments of turbine survival are critical for evaluating turbine operations, dam passage survival, and for assessment prior to and after turbine replacement, to determine the effect of turbine operation and design on survival.

A new tag was developed to provide unbiased estimates of survival for turbine-passed fish. Deng et al. [7] developed a neutrally buoyant externally attached acoustic transmitter (based on the Juvenile Salmon Acoustic Telemetry System (JSATS; [13]) to eliminate pressure related biases in turbine survival estimates. This neutrally buoyant externally attached acoustic transmitter adds no excess mass to the fish and occupies no space within the coelom, and thus has an effective tag burden of zero. Therefore, this new tag, designed specifically for short-term studies of turbine survival, could improve the accuracy of survival estimates for turbine-passed fish.

Extensive laboratory testing was conducted prior to field testing the new externally attached tag. Initially Deng et al. [7] validated that the manufacturing process (i.e., encasing the negatively buoyant JSATS tag in a positively buoyant shell) did not affect the acoustic properties of the transmitter. Further research confirmed that the presence of the tag did not affect fish's performance compared to untagged individuals. For example, compared to untagged fish, there were no differences in growth or mortality over a 14-d holding period, or in predator avoidance [7], [14]. When evaluating swimming performance, juvenile Chinook salmon tagged with the neutrally buoyant external transmitters had a lower critical swimming speed (*Ucrit*) than untagged individuals. However, when compared to fish internally implanted with an acoustic transmitter and a passive integrated transponder (PIT) tag (a combined mass of 0.53 g), there was no significant difference in *Ucrit* [14]. Further, no mortalities or tag loss were observed during exposure to shear forces [7] and the presence of the tag did not increase the fish's susceptibility to barotrauma when compared to untagged fish [15].

Despite these positive results, few studies have assessed tag effects associated with external attachment in the field (reviewed in [16]). Literature also suggests that external tagging can be invasive [17] and can affect overall performance [18], swimming ability [19], [20] and susceptibility to predation [21]. Indeed, a concern of fisheries managers within the US Pacific Northwest is that predation rates may be higher in the field than those observed in the laboratory as a protruding tag may attract avian and aquatic predators. If predation is elevated due to the presence of an external transmitter, any reduction in bias to survival estimates due to barotrauma could be offset by biases specific to the external tag. Therefore, although preliminary laboratory results suggest this new tag may be an effective tool for estimating survival in turbine-passed fish, a field study was required to ensure the presence of an external transmitter would not reduce survival or affect behavior under field conditions. The field study compared survival and travel time between fish tagged with neutrally buoyant external transmitters and those implanted internally with acoustic transmitters.

The objectives of this study were to compare survival and travel times of juvenile Chinook salmon internally and externally JSATS-tagged as well as evaluate loss of external transmitters and examine injury in externally tagged fish. Similar estimates of survival and travel time between tag types would determine if external tags negatively influence performance relative to internally tagged fish and could be useful for turbine passage studies (although passage of turbines was not evaluated during this study). Therefore, the null hypothesis of no significant difference in survival or travel time attributed to tag type was tested. With respect to tag loss, we predict results to be similar to laboratory studies which indicated retention of the neutrally-buoyant external tags for at least 13 days [7].

**Figure 1:**
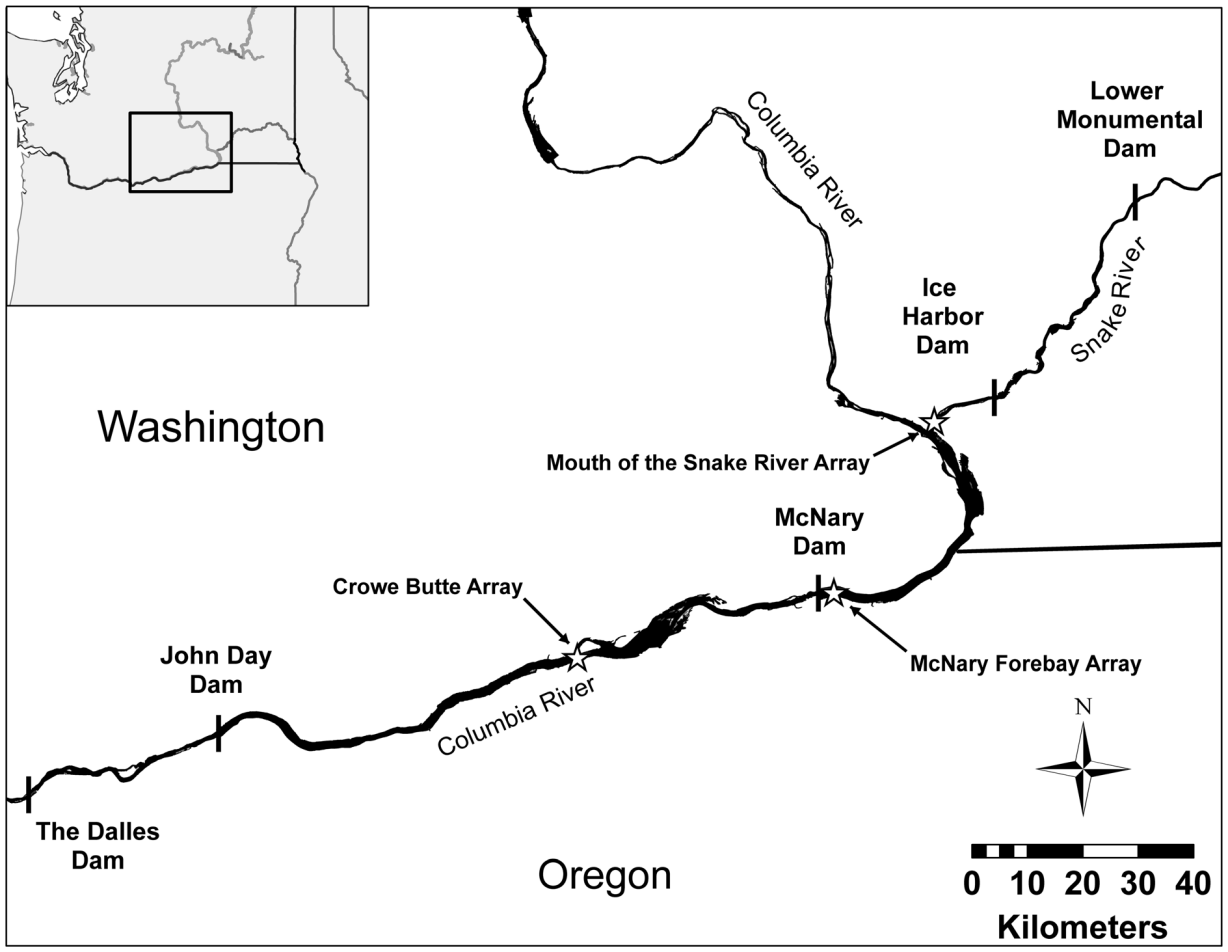
Fish were tagged at Lower Monumental dam and released in either the tailrace (fish with active tags) or the forebay (fish with dummy tags) of Ice Harbor Dam. Survival for fish with active tags was calculated downstream to an array at the mouth of the Snake River (12 km downstream) and to the McNary Dam forebay (65 km downstream). Crowe Butte was used as a downstream array to calculate probabilities of survival and detection. Dummy tagged fish were recaptured using the separation-by-code system at the McNary Dam juvenile bypass system.

Tagged (approximately half with each of the two tag types) hatchery subyearling Chinook salmon were released in the tailrace of Ice Harbor Dam, located on the Snake River just upstream of its confluence with the Columbia River (538 river kilometers [rkm] upstream from the mouth of the Columbia River; Figure 1). We monitored post-release behavior and survival of individuals while migrating downstream to detection arrays at the mouth of the Snake River and at the forebay of McNary Dam in the Columbia River. This region of the Columbia River Basin was chosen as it is known to have high levels of predation, especially bird predation [22], [23]. Additionally, a separate group of fish was tagged with dummy neutrally buoyant external tags and an internally implanted PIT tag and was recaptured at the McNary Dam juvenile bypass facility for examination of tag retention and tissue response. A high occurrence of tag loss or major injury due to the presence of an external tag would also bias survival estimates and would consequently compromise any benefit of eliminating bias due to tag burden in internally tagged fish that passed through turbines.

## Materials and Methods

### Ethics statement

Holding conditions and all experimental procedures were approved by the Institutional Animal Care and Use Committee (IACUC) of the Pacific Northwest National Laboratory (PNNL).

#### 2.1. Fish Acquisition and Holding

Subyearling fall Chinook salmon *Oncorhynchus tshawytscha* were obtained from Lyon's Ferry Fish Hatchery (operated by Washington Department of Fish and Wildlife) and transported to Lower Monumental Dam on May 29, 2012 for holding. Subyearling fish were used as they are the smallest class of seaward-migrating juvenile salmonid smolts and thus represent the class of fish that is most likely to be susceptible to the negative effects of tag presence (i.e. tag effects related to tag burden). During the study period, all fish were held at the juvenile fish facility (JFF) of Lower Monumental Dam (located in the Snake River, 589 rkm upstream of the Columbia River mouth) in three 650-L circular tanks supplied with 13.9–17.7°C flow-through river water. Fish were fed to satiation with 1.5-mm Bio Vita Fry (Bio-Oregon, Longview, Washington) every second day. Fish selected for tagging were restricted from feeding for 24 h prior to tagging. Since fish were fed every second day, this could have led to variance up to 24 h in the amount of time feed was withheld prior to tagging. Lower Monumental Dam was chosen as a holding and tagging site as facilities were already in place for concurrent tagging studies.

#### 2.2. Surgery

**Figure 2:**
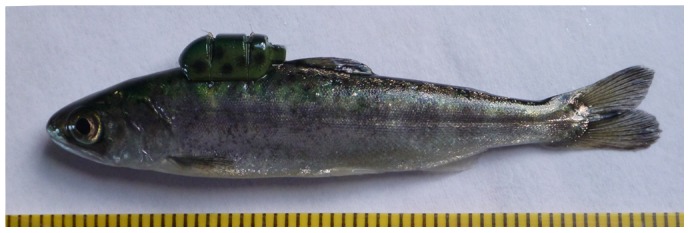
A juvenile Chinook salmon with an externally attached, neutrally buoyant acoustic transmitter.

During surgery, an 80-mg/L solution of tricaine methanesulfonate (MS-222) buffered with 80-mg/L solution of sodium bicarbonate was used to anesthetize the fish until they reached stage 4 anesthesia (as described by [24]). The fork length (FL; mm) and mass (g) of each fish were measured before being assigned to a surgeon and tag type. For external attachment of tags, fish were placed on a foam rubber pad and oriented dorsal side up (for detailed methods and images of tags, see [7], [15]). The neutrally buoyant acoustic transmitters were attached to the dorsal musculature anterior to the dorsal fin by two simple interrupted sutures (Monocryl 5-0 absorbable monofilament) using 2×2×2×2 reinforced square knots (similar to Deters et al. [25]). The sutures rested in two grooves on the top of the transmitter (Figure 2). For internal implantation of tags, fish were placed ventral side up on a foam rubber pad. Internal transmitters were surgically implanted by making a 5- to 6-mm incision on the linea alba, inserting a JSATS tag, and closing the incision with two simple interrupted sutures (Monocryl 5-0 absorbable monofilament) using 1×1×1×1 reinforced square knots (similar to Panther et al. [26]; Deters et al. [25]). For both tag types, a small tube was inserted into the fish's mouth during tagging to provide a constant maintenance flow of 40-mg/L MS-222 buffered with 40-mg/L sodium bicarbonate. When tagging was completed, researchers placed the fish in 20-L perforated buckets that were held within a larger tank of fresh water. Once a bucket contained 10 tagged fish, it was transferred to a 680-L double-wall insulated transport tank, with flow-through water until transportation.

Fish were tagged over 13 d from June 10 through June 25, 2012. External and internal tagging occurred simultaneously. One surgeon performed all external surgeries. As internal implantation is more time consuming than externally attaching tags (see Deng et al. [7] for examples of tagging time differences), two surgeons were required in one tagging session for internal implantation of tags. Surgeons worked in teams of two while alternating days; thus, four expert surgeons conducted internal surgeries. These surgeons all received identical and extensive surgeon training (using concepts presented by Deters et al. [27] and Cooke et al. [28]) and had similar levels of expertise. Transmitters used for surgical implantation were the JSATS micro transmitters designed by Advanced Telemetry Systems (Isanti, Minnesota; third-generation 2012 model). Internal transmitters were 10.79 mm long, 5.26 mm wide, and 3.44 mm thick and weighed 0.304 g in air and 0.186 g in water. External tags were comprised of the same JSATS transmitter that was used for the internally tagged fish but were encased in a positively buoyant substance. The external tags were manufactured using the protocols for the “Type A” tag described in Deng et al. [7] except were created using an epoxy mixture density of 0.72 g/cm^3^ instead of the 0.68 g/cm^3^ density used by Deng et al. [7]. The resulting weight in water of the tags was 0.028 g. Both internal and external tags had a single model 337 battery that pulsed every 3 s, yielding a tag life of approximately 24 d. The combined mass of the JSATS transmitter and encasement was formed to be slightly negatively buoyant to reduce the likelihood that the external tags would be detected at downstream detection arrays if they became separated from a fish.

A total of 490 hatchery-reared juvenile Chinook salmon were tagged and 487 were released (244 internal and 243 external) for the first phase of field research. Twenty fish of each tagging treatment were tagged during the first 12 days while five of each treatment were tagged the last day; three fish died overnight following tagging (two external and one internal). Only fish 95 mm or greater were tagged; thus, fish size ranged from 95 to 118 mm fork length (FL; mean ± standard error [SE] = 101.2±0.2 mm). Fish size is noteworthy, as the study fish were considerably smaller than run-of-river fish collected by the smolt monitoring program (SMP), a multiagency initiative conducted each year throughout the lower Snake and Columbia Rivers to sample the general characteristics of seaward-migrating salmonids [29]. According to Fish Passage Center data for 2011, taggable fish (i.e., ≥95 mm FL) collected by the Lower Monumental JFF during our study period ranged from 95 to 142 mm FL (mean ±SE = 106.7±0.2 mm).

#### 2.3 Releases

Prior to tagging, 10 dead fish with active external tags attached and 10 unattached active external tags were released in the tailrace of Ice Harbor Dam. This was done to ensure that if a fish died or lost a tag, detection would not occur at detection arrays 12 km downstream. None of the tag codes from these initial releases were detected downstream, thus indicating minimal probability of dead tagged fish or a tag separated from a fish would be confused for a detection of a live fish.

Immediately upon completion of tagging for each day, fish were transported to Ice Harbor Dam by truck in an aerated 680-L double-wall insulated transport tank. Buckets were held at the Ice Harbor Dam JFF in a 650-L circular tank with flow through river water overnight prior to release. Immediately prior to release, we confirmed that all transmitters were operational. Buckets were then placed in the transport tote and transported from the JFF to the powerhouse tailrace at Ice Harbor Dam (approximately 5 min). After an examination for any overnight mortality, all fish were released into the tailrace through a PVC flex hose (12.2 m long and 5.1 cm in diameter) with an attached receiver funnel comprised of PVC reducing couplers. Fish were released at the outlet of hydroturbine Unit 1 in the powerhouse tailrace. None of the fish were released at the inlet to the turbine. Directly examining turbine survival through the turbines would require a more complex study design to ensure fish were properly acclimated to represent fish approaching the dam [4]. This type of research is planned for future years and was not within the scope of this research.

Acoustic transmissions from tagged fish were detected and decoded by JSATS autonomous receivers (Model SR5000; Advanced Telemetry Systems, Inc., Isanti, MN, USA). Receivers were deployed in lines (referred to as “arrays”) that ran approximately perpendicular to shore (see Titzler et al. [30] for deployment methods). Arrays were located near the confluence of the Snake and Columbia rivers (the “mouth of the Snake” array, 12 rkm from Ice Harbor Dam and 525 rkm from the mouth of the Columbia River), in the forebay of McNary Dam (472 rkm from the mouth of the Columbia River), and at Crow Butte (422 rkm from the mouth of the Columbia River; Figure 1).

#### 2.4. Evaluation of Tag Retention

To evaluate retention of the external transmitters, an additional 480 juvenile Chinook salmon were tagged with PIT tags and dummy external transmitters on June 28 and 29, 2012. Fish used ranged from 92 to 122 mm in fork length (mean ±SE = 100.9±0.3 mm). Fish were tagged with an externally attached neutrally buoyant dummy transmitter and an internally injected PIT tag (Destron Technologies, St. Paul, Minnesota). The coating material, dimensions and weight of the dummy tags were the same as the functional external tags. PIT tags were 12.5×2.1 mm, 0.10 g in air, and 0.06 g in water.

Surgical methods (e.g., anesthesia time, equipment, attachment methods) were the same as for the survival study, and surgeries were conducted by the same surgeon. All PIT tagging occurred prior to affixing the external dummy tag and was completed by one tagger. PIT tags were implanted using a 12-gauge hypodermic needle and syringe just posterior to where the tip of the pectoral fin lies against the body and slightly dorsal of the linea alba. The needle was inserted at an angle of about 30° pointing toward the posterior of the fish. As with the survival study, the fish were placed in buckets upon completion of surgery for recovery and transportation.

After surgery, buckets were placed in 650-L circular tanks with flow-through river water for 16–24 h at Lower Monumental Dam. Following this recovery period, buckets were transported to the Ice Harbor Dam forebay boat launch and boated to the forebay of Ice Harbor Dam where the fish were released ∼500 m upstream of the dam. Fish were released in two groups of 240 fish on June 29 and 30, 2012. Releases occurred in the forebay to allow for dam passage prior to recapture and evaluation. Including dam passage in the tag retention evaluation exposed fish to environmental conditions that externally tagged fish may experience during a turbine passage survival study.

Fish were PIT tagged so that those entering the JFF at McNary Dam could be later examined following detection by the separation-by-code system (SbyC). Upon entering the JFF, juvenile salmonids and other small fish drop through the separator bars and are directed from the trough under these bars to a flume which carries them past PIT tag detector coils to a rotating gate [31]. This gate is normally open to pass fish back to the river but can rotate to separate out and collect PIT-tagged fish.

Fish that entered the JFF at McNary Dam were collected from July 1 to August 15, 2012 using the SbyC. Collected fish were routed to a holding tank and examined the following day. Fish were evaluated on six criteria similar to Deng et al. [7]: (1) presence of the external tag, (2) whether sutures were loose, untied, or lost, (3) the extent of tissue tearing (i.e. the length of the longest flesh tear caused by a suture tearing the tissue; measured in millimeters), (4) tag indentation, where the tag leaves an imprint in the flesh (classified as absent, mild or severe); (5) percentage of tissue laceration, caused by the tag rubbing against the tissue (classified as absent, mild or severe); and (6) percentage of discoloration beneath the tag (classified as not present, <50% of the tag surface, or >50% of the tag surface).

#### 2.5. Statistical Analyses

Differences in survival and travel time between groups from release to the array near the confluence of the Snake and Columbia rivers as well as from release to the McNary Dam forebay array were determined. Because there was concern that the material and geometry of the external tags may have compromised detection probability due to the modifications required to achieve neutral buoyancy, detection probabilities were also compared between externally and internally tagged groups. The data were initially explored for any bias caused by the experimental design such as surgeon-specific survival differences (used for internal implantation when more than one surgeon was used) and differences in fish size distributions between treatments.

Probabilities of survival and detection (i.e., the probability of survival, migration, and tag function and retention) were estimated with R (version 2.15.1) using the single release-recapture model (hereafter referred to as the CJS model; [32], [33], [34]). The CJS model requires detections at the point of interest as well as at one downstream site. Therefore, the CJS model required the use of the Crow Butte array downstream of McNary Dam to estimate survival to the forebay of McNary Dam. *F*-tests were used to compare survival by treatment to both the mouth of the Snake River and the McNary Dam forebay arrays.

Travel time was calculated as the difference between release and first detection at the mouth of the Snake River array and between release and first detection at the McNary Dam forebay array. Travel rate (km/day) was then determined by dividing the distance traveled by the travel time. Because travel times typically have a right-skewed distribution, all data were subjected to a natural log (ln) transformation prior to analysis. Using the response variable ln (travel time), ANOVA tables were constructed to test for differences in tag type to both the mouth of the Snake River and McNary Dam forebay arrays. Because study fish were much smaller than run-of-river fish (a mix of wild fish and hatchery fish not recently released into the river) and consequently may have been more likely to be subject to tag effects, data were explored for a length effect. Regression analyses were used to determine relationships between length and ln (travel time) to the McNary Dam forebay array. For internally tagged fish, the same analyses were repeated but with tag burden instead of length.

Detection probability was estimated using the CJS model. Detection probability was calculated by comparing the number of fish detected at a point of interest (mouth of the Snake River and the McNary Dam forebay arrays) to the number of fish detected at a downstream array (McNary Dam forebay and Crow Butte arrays, respectively). When calculating detection probability, two things need to be determined. First, the number of fish detected at the array of interest and at a downstream array. Second, the number of fish only detected at a downstream array and not at the array of interest. With this information, the probability of detection at the array of interest can be determined.

## Results

Distributions of fish size did not differ significantly (P = 0.6) between internally and externally tagged fish. There were no significant (P = 0.5) differences in survival to the McNary Dam forebay array among the four surgeons internally implanting tags. Detection probability was 100% for both internally and externally tagged fish at both the mouth of the Snake River and McNary Dam forebay arrays.

### 3.1. Survival Estimates

**Figure 3:**
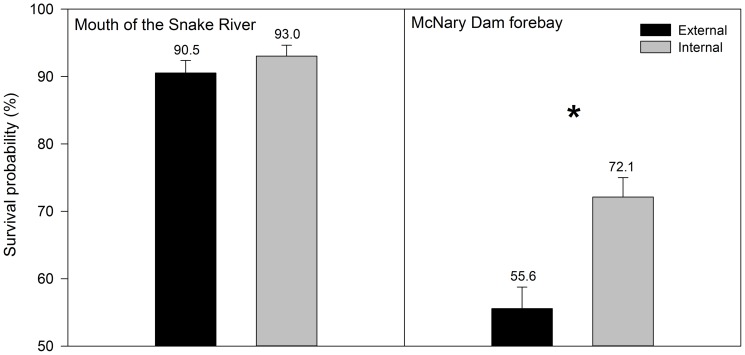
Survival probability (with SE indicated by error bars) to the mouth of the Snake River and to the McNary Dam forebay for subyearling Chinook salmon with two different tag types (internally implanted JSATS tag and externally attached neutrally buoyant JSATS tag) released in the tailrace of Ice Harbor Dam in the Snake River, Washington, in June 2012.

There were no significant differences (P = 0.3) in survival probability (mean [%]±SE) to the first detection array at the mouth of the Snake River, 12 rkm downstream from release (Internal: 93.0±1.63; External: 90.5±1.88; Figure 3). Survival to the McNary Dam forebay array (65 rkm downstream from release) was considerably lower than survival to the array at the mouth of the Snake River and there was a significant difference between groups (P<0.0001; Figure 3). Survival estimates (mean [%]±SE) were higher for internally tagged fish (72.1±2. 87) than for externally tagged fish (55.6±3. 19).

### 3.2. Travel Time

**Figure 4:**
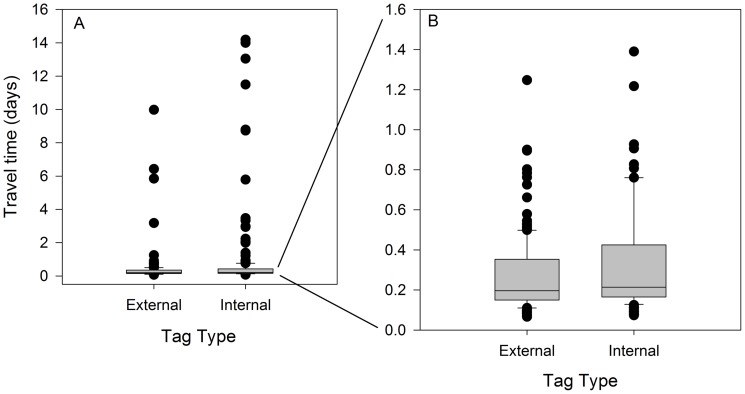
Travel time (days) of subyearling Chinook salmon with two different tag types (internally implanted JSATS tag and externally attached neutrally buoyant JSATS tag) to the mouth of the Snake River (12 rkm downstream) from the tailrace of Ice Harbor Dam in the Snake River, Washington. Travel time was very right-skewed with many outliers (A), data from the first 1.6 days are magnified in panel B, to allow data from the majority of fish to be better visualized (line within box  =  median; lower and upper edges of boxes  = 25th and 75th percentile, respectively; ends of whiskers  = 1.5× interquartile range; points  =  outliers).

There was a large amount of variability in the time it took fish to travel the short distance from the release location to the array near the mouth of the Snake River. Travel times ranged from 0.07 d to over 2 weeks while individual travel rates ranged from 0.85 to 180 km/d (Figure 4). Although differences in travel time with tag type were significant (P = 0.002), they appeared to be largely driven by the many outliers and are likely not biologically relevant as median travel times (days ±SE) were very similar between internally (0.21 d±0.1) and externally tagged fish (0.20 d±0.06; Figure 4). However, there was considerably more variability among travel times for internally tagged fish than externally tagged fish.

**Figure 5:**
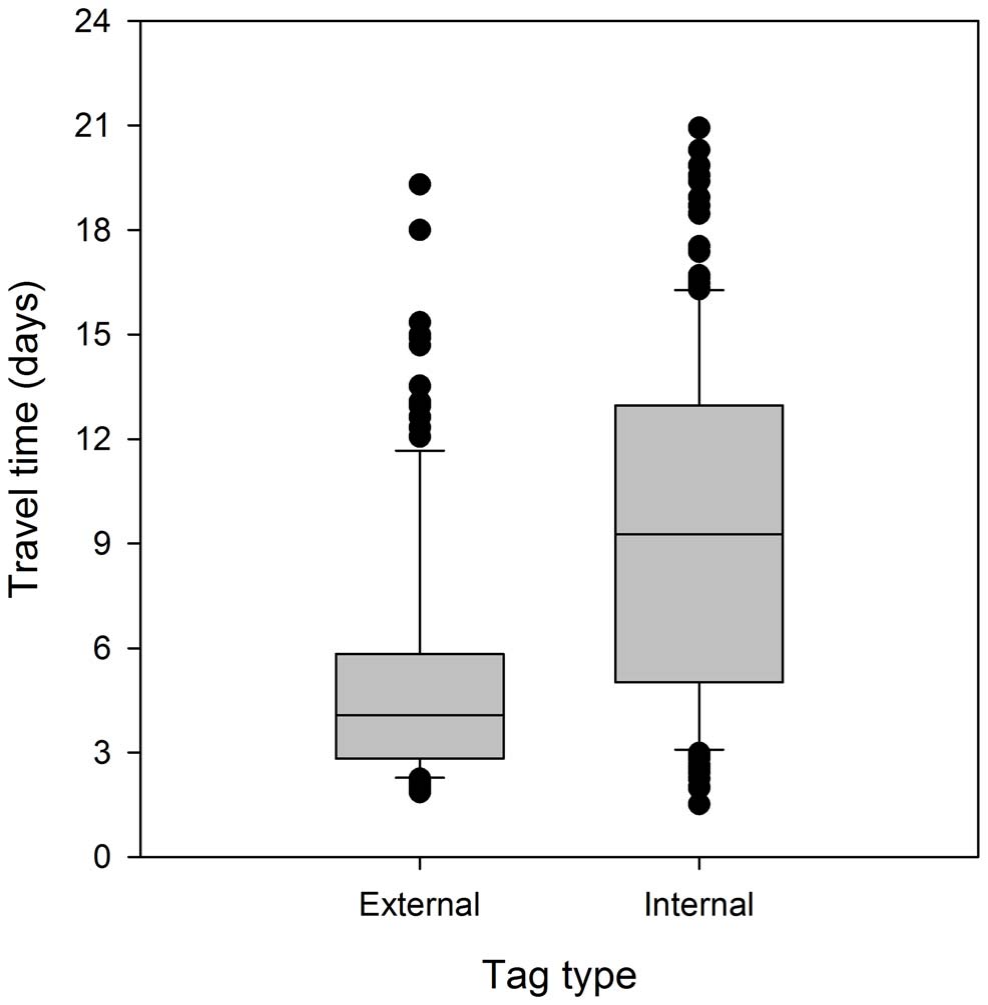
Travel time (days) of subyearling Chinook salmon with two different tag types (internally implanted JSATS tag and externally attached neutrally buoyant JSATS tag) to McNary Dam forebay in the Columbia River (65 km downstream) from the tailrace of Ice Harbor Dam in the Snake River, Washington (line within box  =  median; lower and upper edges of boxes  = 25th and 75th percentile, respectively; ends of whiskers  = 1.5× interquartile range; points  =  outliers).

Similar to the trends observed to the first array, travel time to McNary Dam forebay, 65 rkm downstream, was highly variable among individuals. Travel times ranged from 1.5 d to almost 3 weeks while travel rates ranged from 3.1 to 43.1 km/d. There were also significant differences (P<0.0001) between groups. However, unlike to the mouth of the Snake River, there were large differences in travel time estimates (days ±SE) between tag types where estimates for externally tagged fish (4.1 d±0.3) were more than twice as fast as those for internally tagged fish (9.3 d±0.4; Figure 5).

**Figure 6:**
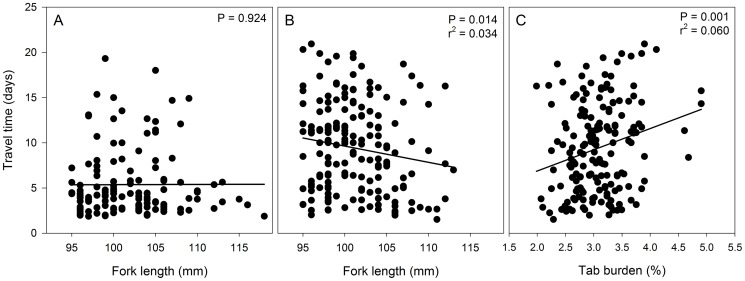
Relationships between size (panel A and B) and tag burden (panel C) with travel time to the array in the McNary Dam forebay for subyearling Chinook salmon either tagged with externally attached neutrally buoyant JSATS tag or an internally implanted JSATS tag.

There was a significant yet rather weak correlation between fish length and travel time among internally tagged fish (P = 0.014; r^2^ = 0.034) but not among externally tagged fish (P = 0.9; Figure 6). Fish tagged with the neutrally buoyant external tag had essentially no tag burden while tag burdens for internally tagged fish ranged from 2.0% to 4.9% (mean ±SE = 3.1±0.03%). Among internally tagged fish, the correlation coefficient from the relationship between tag burden and travel time was greater (P = 0.001; r^2^ = 0.060; Figure 6) than that from the relationship between travel time and length indicating that smaller fish with higher tag burdens traveled slower than larger fish with lower tag burdens.

### 3.3. Tag Retention to McNary Dam

**Figure 7:**
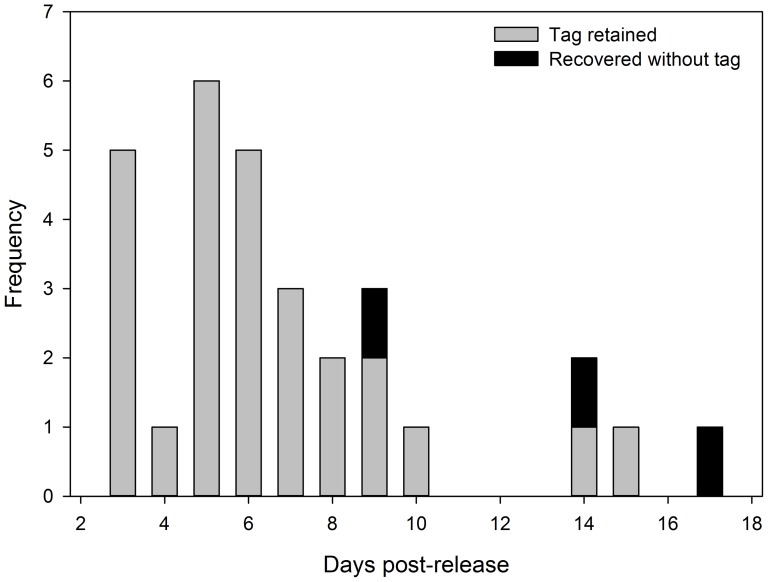
The number of subyearling Chinook salmon affixed with dummy neutrally buoyant external tags and implanted with PIT tags that were recovered using the separation-by-code system at the McNary Dam juvenile fish facility. Fish were released across two days in June 2012 in the forebay of Ice Harbor Dam in the Snake River, Washington.

Thirty (6.3%) of the released 480 dummy tagged fish were recovered using the SbyC at McNary Dam. Fish were recovered between 3 and 17 d post release; three (10% of those recaptured) were recovered without their external transmitters attached (Figure 7). The first fish without a tag was recovered 9 d after release, and although the tag was missing, the posterior suture was retained. The second fish was recovered 14 d after release. This fish was dead and was missing not only the tag but both sutures. The third fish was recovered 17 d after release and was missing the tag and both sutures. On this same day, two tags were also recovered in the holding tank. Dummy-tags did not have an individual marking and therefore we could not determine which fish these tags came from.

Many tags were loose upon recovery (19% of fish recovered with tags) and tearing was commonly present at the suture insertion point. All fish had some degree of tissue tearing. The longest tear measured on individual fish ranged from 1 to 10 mm (median ±SE = 2.0±0.41 mm). However, only two fish had tears greater than 5 mm (they both had 10 mm tears) and both were found without tags. For these fish, the longest tear measurement was made from suture entry point to suture exit point across the dorsal area of the fish since the suture was likely ripped out when the tag was lost. Fish condition upon recovery was highly variable. Most fish had discoloration at the tag site (the color being lighter under the tag); 46.7% of fish recovered had discoloration on less than 50% of the area under the tag while 36.7% of fish had discoloration on more than 50% of the area under the tag (Table 1). Only 16.7% of fish recovered had no discoloration. Tissue laceration, caused by the tag rubbing against the tissue, was present in 80% of fish (Table 1). Tag indentation was not as common; 50% of the fish observed had no tag indentation and most others (46.7%) had mild tag indentation (Table 1).

**Table 1:** Injuries observed in 30 subyearling Chinook salmon affixed with dummy neutrally buoyant external tags and implanted with PIT tags recovered by the separation by code system at the McNary Dam Juvenile Fish Facility.

	Proportion of sample (%)		
	None	Mild	Severe
Discoloration	16.7	46.7	36.7
Tissue laceration	20.0	63.3	16.7
Tag indentation	50.0	46.7	3.3

Fish were released on June 29 and 30, 2012 in the forebay of Ice Harbor Dam in the Snake River, Washington.

## Discussion

### 4.1. Survival

The null hypothesis of no significant difference in survival with tag type from the tailrace of Ice Harbor Dam to a detection array 12 km downstream was supported, despite fish passing through an area where high predation was expected. A short distance downstream of the confluence of the Snake and Columbia rivers, there are three islands: Foundation, Badger, and Crescent. These islands are known breeding sites for large colonies of double-crested cormorants *Phalacrocorax auritus*, American white pelicans *Pelecanus erythrorhynchos*, and Caspian terns *Hydroprogne caspia*. The double-crested cormorant colony of Foundation Island and the Caspian tern colony of Crescent Island consumed an estimated one million juvenile salmonids annually from 2004 to 2009 [22]. Further, the Caspian terns nesting on Crescent Island are estimated to have the highest per capita predation rate on PIT-tagged juvenile salmonids of all studied bird colonies during 2007 to 2010 [23].

Further indication that the reach upstream of McNary Dam has high predation was the relatively low survival (28%–30%) noted by McMichael et al. [35] for PIT tagged subyearling Chinook salmon released in the Hanford Reach (located in the Columbia River, 639 rkm from the mouth) and detected downstream at McNary Dam. The authors suggested that this mortality was due to high numbers of smallmouth bass *Micropterus dolomieu* as well as Caspian terns. Indeed, not only are populations of smallmouth bass in the lower Snake River relatively large compared to populations in the Columbia River [36], but smallmouth bass tend to prey more upon smaller subyearling salmon over yearling size salmonids [37], [38], [39], [40].

Although the external tag did not have any negative effects on survival relative to the internal tag during the first array 12 km downstream from the release site, survival estimates from release to McNary Dam (65 km downstream) indicate that it is not likely useful for longer-term projects as survival was considerably lower for externally tagged fish. However, it is clear from the loss of external tags among fish recaptured in the McNary SbyC that tag retention compromised results of survival estimates to the forebay of McNary Dam. Thus, in the reach between the mouth of the Snake River and McNary Dam, comparisons of survival between internally and externally tagged fish likely do not reflect true survival or provide insight on possible susceptibility to predation.

### 4.2. Tag retention and injury

Given low sample sizes of recovered fish, it is difficult to make firm conclusions about the retention of the external transmitters. We documented a tag loss of 10% in fish recovered at McNary Dam; external transmitters were noted to be shed upon recapture between approximately 9 and 17 d after release, although the tags could have been lost before their recapture at McNary Dam. This indicates that tag loss influenced data to McNary Dam and possibly to a lesser extent to the mouth of the Snake River. The loss of tags observed in this field trial was not surprising considering results of the laboratory evaluation of this tag [7] and other laboratory studies of suture retention (e.g., [25], [26]). In the laboratory study of the external neutrally buoyant transmitter, tag loss occurred at 13 d after tagging; one fish, or 4.8% of test fish, shed its tag during the 14-d holding experiment [7]. Although losses may be expected sooner in a more dynamic river environment and when passing hydro facilities or juvenile bypass systems, there was no loss of external transmitters during laboratory shear tests where fish were exposed to water jet velocities up to 12.2 m/s [7]. There was also no tag loss during the 4 days that the fish were held following shear tests. These laboratory results therefore suggest that immediate tag loss should not be an issue.

All tags in this field evaluation were sutured to the fish using 5-0 Monocryl absorbable monofilament sutures. Deters et al. [25] suggested that these absorbable sutures are not necessarily absorbed but actively expelled by the fish (physiologically) or passively expelled due to drag on the suture. They found that the sutures typically moved closer to the incision with healing while the suture and knot remained intact. Such a migration of the sutures would cause loosening of external transmitters over time, leading to eventual loss. Panther et al. [26] also documented loss of sutures over a 98-d holding period. They found that juvenile salmonids held in warmer water (20°C) had faster suture loss than fish held in cooler (12°C) water. This may equate to higher tag retention in colder water than what was observed during this study (14–18°C). Similar temperature-dependent suture loss was found in juvenile Chinook salmon [27] and in white bass [40]. Although we cannot precisely quantify a percentage of total tag loss, our data in combination with laboratory results suggest that tag loss is a concern for these external tags after approximately 8 d. However, the external tags were not designed to stay on the fish long term but just long enough to conduct a single dam turbine survival study, which would typically be less than 7 d. Because fish known to have lost their dummy external tags were recovered at McNary Dam 9, 14, and 17 d after their release, this does not disregard the viability of the tags for the types of studies for which they were intended.

Of fish recovered at McNary Dam with the tag still affixed, sutures were commonly loose upon recovery. This could be due to dissolving of the sutures, suture stretching, tissue tearing at the point of insertion, or natural migration of sutures out of the fish's body. Tissue tearing was the most common injury observed in recovered fish (tears ≥1.5 mm seen in 83% of fish). As sutures loosen, the tag separates from the body and swimming with this loosened tag would likely accelerate tissue tearing as the tag moves away from the body. Similarly, Morgan and Roberts [41] found that strong exercise intensified problems associated with external tagging and described long-term difficulties resulting from incomplete healing of wounds. However, the use of glue to fix the tag to the sutures could yield higher tag retention during future research.

As the number of days post-release progressed and tags separated from the body, we generally observed a decline in the condition of recaptured fish. The percutaneous nature of attaching external tags can cause muscle damage and dorsal scale loss in the vicinity of attachment [18] as well as open wounds that cause histopathological damage and infection [17]. For these reasons, the tissue tearing observed in this study is a concern for long term survival. However, tissue tearing may not affect performance in the short term and other injuries were generally minor. Discoloration at the tag site was common where the skin was lighter under the tag but it is improbable that this discoloration influences overall fish health or condition. Observations of external condition suggest that injury due to tag presence likely did not affect fish performance within the first week. However, observations of deteriorating condition combined with the known long-term histopathological effects of external tagging (e.g., [17]) suggest that even if a tag is retained beyond the first week, tag effects could manifest and results would be unreliable.

### 4.3. Travel Time

Overall, travel times of fish tagged for this study were relatively slow. The 65-rkm distance from the mouth of the Snake River to McNary dam was covered in a median of 5.7 d by the hatchery-origin fish used in this study compared to a median of approximately 2 d for a concurrent study on run-of-river wild subyearling Chinook salmon in the same river reach of the Columbia River basin (Geoff McMichael, Pacific Northwest National Laboratory, unpublished data, 2012). Slower travel times in fish recently released from hatcheries are common [42], [43], [44]. For example, travel rates of wild juvenile steelhead were found to be twice as fast as hatchery-reared steelhead and the hatchery-reared fish were observed moving upstream after release, delaying their seaward migration [43]. Behavioral and physiological changes occur in juvenile salmonids during the smoltification period cueing migration to begin. As these cues are regulated by environmental conditions [42], the unnatural rearing conditions of hatcheries produce hatchery-sourced fish that are often not physiologically cued for migration upon release [45], [46]. However, even upon release, some in-river migration experience may be required to initiate physiological changes accompanying smoltification [47], [42]. By using hatchery fish in this study, travel times were much slower than would be expected if the study were conducted on run-of-river fish which would have been actively migrating seaward.

There was large variability in travel times between internally and externally tagged fish. Statistically significant differences in travel time to the mouth of the Snake River were driven by lingering individuals; although individual travel times were variable, median travel times for both groups were approximately 0.2 d over this 12 km reach. To the forebay of McNary Dam, however, large differences in travel time were apparent whereby estimates of travel time for externally tagged fish were more than twice as fast as those of internally tagged fish. Reasons for this difference in travel time with tag type are ambiguous. Tag loss is a factor to consider as it is possible that externally tagged fish lingered as much as internally tagged fish but were not detected at later dates because their tags were shed (similar to fish recaptured at McNary Dam 9-17 d after release). In addition, the relationship between tag burden and travel time in internally tagged fish suggests the presence of length-specific tag effects. It is common for smaller seaward-migrating salmonids to have slower rates of travel [48], [44] and consequently, those smaller fish have reduced survival [49].

### 4.4. Efficacy of the Neutrally Buoyant External Tag

No observed statistical differences in survival with tag type to the mouth of the Snake suggest the externally attached neutrally buoyant JSATS transmitter may be a viable option for examining turbine survival, even in areas with high predation. Dummy tag recoveries revealed high tag retention within the first week of the study, and only minor external tag-related injuries were observed during this period among fish recaptured at McNary Dam. However, tag loss and injury results from this study beyond the first week of study reveal that the tag is only appropriate for short-term turbine passage survival studies when survival estimates can be obtained in ∼8 d or less. For route specific survival estimates, such as turbine survival studies, a minimum of two downstream detection points are required and downstream arrays are required to be located as far away from release as a dead fish with a transmitter could passively drift ([50]; in this study, dead fish with external neutrally buoyant tags attached were not detected 12 km downstream). Migration rates in the Columbia River Basin (CRB) vary with species, length and flow characteristics [48] but the literature suggests that seaward-migrating juvenile salmonids cover sufficient distance for a turbine passage study to be conducted in less than 7 days. For example Giorgi et al. [48] reported median travel rates of juvenile sockeye salmon and steelhead in the Columbia River across several years of data to be 21.5±8.4 km/d and 30.4±10.9 km/d, respectively. Over a shorter distance (i.e., within the Snake River Lower Granite Dam reservoir; 643–695 rkm upstream from the mouth of the Columbia River), migration rates of juvenile spring Chinook salmon ranged from 3.08 to 12.38 km/d [51]. Thus, it is reasonable to design a short-term turbine survival study using this new tagging technology and expect valid results before tag loss occurs.

Although the tag may be appropriate for research over relatively short reaches, we did observe reduced survival in externally tagged fish and large differences in travel time over a longer term. There are confounding factors to consider, such as a known tag loss and possible length-specific tag effects in internally tagged fish. Despite this however, the results to McNary Dam could also indicate that external tags negatively influence fish performance more than internally implanted tags.

Results of previous laboratory research suggest that the use of a neutrally buoyant externally attached tag may alleviate the bias encountered in turbine-passage survival studies when using internal tags (e.g., [7], [14], [15]) and this field research suggests that carrying an externally attached transmitter may not increase predation compared to carrying an internally implanted transmitter during such studies. Many aging turbines in the Lower Snake and Columbia river dams will soon be replaced with turbines designed for safer fish passage. For example, a test turbine runner is scheduled to be installed at Ice Harbor Dam and will be the first of its kind designed with an innovative process for improved fish passage [52] that incorporates recent research on relationships between pressure exposures and mortality [4], [53]. An appropriate application of this new external tagging technology would be to compare older turbines to their replacements. Without appropriate knowledge of baseline turbine survival, accurately determining the benefits of new turbine designs may not be possible.

We recommend further research using a smaller external transmitter and run-of-river fish to examine survival estimates of turbine passed fish. Research is currently under way at PNNL to produce a smaller, possibly injectable JSATS transmitter [54], [55] that if used, could reduce the volume of the external transmitter by approximately 40%. Further volume reduction could be attained by using a smaller battery with a reduced life (7–10 d). This could also enhance tag retention because drag or the likelihood of snagging on debris may be reduced when using a smaller external tag. In addition, reaction to tag attachment procedures of hatchery reared fish can differ from wild fish [56]. In the present study, run-of-river fish were not attainable given their listed status under the Endangered Species Act. However, the future use of run-of-river study fish captured at one of the bypass systems at a Snake or Columbia river dam would provide a more robust ability to examine new turbine design features when implemented on the Columbia River hydropower system.
